# Self-transcendent emotions and global identity in the context of a global threat in Chile: concurrent associations, temporal stability, and longitudinal invariance

**DOI:** 10.3389/fpsyg.2026.1943560

**Published:** 2026-10-12

**Authors:** Lander Méndez, Anna Wlodarczyk, Javier Torres-Vallejos, María José Mera-Lemp, Alberto Amutio

**Affiliations:** 1 Department of Social Psychology, Labor Relations and Social Work Faculty, University of the Basque Country (EHU), Leioa, Spain; 2 Escuela de Psicología, Universidad Católica del Norte, Antofagasta, Chile; 3 Departamento de Psicología Cognitiva, Social y Organizacional, Facultad de Psicología y Logopedia, Universidad de La Laguna, Leioa, Spain; 4 Escuela de Psicología, Facultad de Ciencias Sociales y Educación, Universidad Santo Tomas, Santiago, Chile; 5 Universidad Autónoma de Chile, Temuco, Chile; 6 Facultad de Educación y Ciencias Sociales, Universidad Andres Bello, Santiago, Chile

**Keywords:** perceived threat, self-transcendent emotions, global identity, measurement invariance, global crisis, Chile

## Abstract

The present study analyzed the psychological mechanism by which perceived threat in the face of a global phenomenon influences social identification with superordinate categories, such as global identity, considering the role of self-transcendent emotional experience, measured by self-transcendent emotions. Using a two-wave half-longitudinal design (T1-T2) conducted in Chile (*N* = 224), structural equation modeling (SEM) and path analysis with bootstrapping were applied. The results showed that perceived threat was positively associated with self-transcendent emotions, and that self-transcendent emotions, in turn, was associated with higher global identity levels at both measurement points. The concurrent indirect effects were statistically significant at T1 and T2 (β = 0.21, β = 0.20, respectively); however, the cross-lagged model did not provide evidence of significant prospective effects between perceived threat, self-transcendent emotions, and global identity, once the temporal stability of the constructs was controlled for (*p* >0.05). The longitudinal scalar invariance of the global identity construct (ϕ = 0.67) allowed for the comparison of latent means between both measurements, highlighting a slight but statistically significant decrease in global identification over time (*d* = −0.15, *p* = 0.014). The findings suggest that, although global identity appears to weaken modestly over time as the threatening situation persists, the self-transcendent emotional experience constitutes a resilient psychological mechanism for maintaining identification and a sense of belonging to a human community in the face of global threats.

## 1 Introduction

Social Identity Theory (Tajfel and Turner, 1979) posits that individuals orient a critical portion of their self-concept toward the processes of social categorization and membership in specific social groups. From this analytical framework, social actors seek to establish positive distinctiveness for their ingroup in comparison to relevant outgroups, a process that guides their perceptions, behaviors, and shared norms (Voci, 2006). However, under contemporary conditions of globalization that introduce systemic uncertainty, or when faced with a prolonged perceived exogenous risk, the security of local social identity may be threatened (Ozer, 2020).

Perceived threat refers to a subjective appraisal that an event may produce future harm or loss in relation to personally significant goals or wellbeing; it therefore concerns the meaning attributed to its possible consequences rather than the objective magnitude of the danger itself (Lazarus and Folkman, 1984; Lazarus, 1991). This appraisal may also involve uncertainty regarding the event and individuals' perceived capacity to manage its consequences, and may concern different domains of personal life, including physical security, material conditions, and personally significant values or beliefs (Rousseau and Garcia-Retamero, 2007; Adam-Troian and Bagci, 2021). Such appraisals are not formed in isolation, but are embedded in broader social and cultural frameworks that reflect shared values, symbols, historical experiences, and ideological orientations (Weinstein, 1989). In the context of COVID-19, perceived threat may therefore encompass anticipated harm resulting from infection as well as possible losses affecting social and material conditions (Pizarro et al., 2020, 2024).

Global crises can modify the relative salience of different social affiliations, intensifying the search for security in proximate identities such as the local community or the nation. However, when the threat is perceived as shared and transnational, it may also make human interdependence more visible and activate broader social categories. Within the social identity approach, this does not imply that threat automatically strengthens global identification. Rather, a threat experienced as common to humanity may increase the situational relevance of a superordinate human category, while other contexts may make more proximal identities salient (Adam-Troian and Bagci, 2021). Therefore, the relationship between threat and identity should not be understood solely as a unidirectional process, but also as a situated dynamic dependent on the sociocultural context, the available interpretive frameworks, and the emotional responses that accompany the experience of crisis (see Chiu et al., 2011; Markus and Kitayama, 2010).

### 1.1 Global identity as a superordinate social identification

Superordinate identifications are cognitive and affective constructs that place the focus of self-categorization and group belonging at the highest level of abstraction and inclusion (Turner et al., 1987), encompassing the entire human species or society on a planetary scale (McFarland et al., 2012; Reese et al., 2015). These broad social identifications are diametrically opposed to exclusive intergroup bias (Stone and Crisp, 2007) and necessarily entail the inclusion of all human beings and a genuine concern for their wellbeing, with a focus on the relational aspects of social justice and cohesion (Huo et al., 1996). They represent a reconfiguration of traditional ingroup boundaries (community, nation, ethnicity, religion) toward a global ingroup where the “other” ceases to belong to the outgroup and is included and recategorized as part of a common system (Gaertner and Dovidio, 2000). In this sense, superordinate social identifications are not static psychological constructs; their salience fluctuates according to contextual dynamics and perceived threats (Buchan et al., 2011). Previous studies have shown that their activation can occur in the face of shared threats derived from global phenomena such as climate change or pandemic crises (Reese, 2016), and in self-transcendent affective states with cognitive reappraisal capacity toward global interconnectedness (Stellar et al., 2017).

Different superordinate social entities have been conceptualized in Psychology, such as identification with all humanity, global citizenship, or global identity. While they share the basic component of an inclusive orientation, they have differentiating characteristics and should not be treated as fully equivalent broad social entities, as they activate different cognitive representations and lead to different behavioral outcomes (Carmona et al., 2020). For example, Identification With All Humanity (IWAH) is conceptualized as an individual disposition that encompasses a moral concern (self-investment) and a deep socio-affective bond (self-definition) toward all human beings. It evokes attributes shared by humans as a species and emphasizes the inclusion of all humanity within one's own moral and psychological ingroup (Hamer et al., 2019; McFarland et al., 2012). It directly predicts humanitarian aid (Sparkman and Hamer, 2020; Wang and Xu, 2026) and helps mitigate the dehumanization of outgroups (Bassett and Cleveland, 2019; McFarland et al., 2013).

Global Citizenship, in turn, explicitly incorporates normative and civic components, such as valuing diversity and social justice, and adhering to prosocial values. Based on an ideological-normative scheme, this construct is structured around global awareness (antecedent) and evokes a sense of responsibility to act on collective problems (consequence). It predicts active engagement in systemic change, pro-environmental activism, support for global wealth redistribution policies, and international civic participation (see Reysen and Katzarska-Miller, 2013).

Lastly, Global Identity is understood as a broad form of affective and psychological belonging to a human community or global village, formed from awareness of global problems and interconnectedness with the world as a whole, where individuals assume interdependence and shared responsibility (de Rivera, 2022; Der-Karabetian and Ruiz, 1997). Based on Social Identity Theory and Self Categorization Theory it represents the highest level of abstraction in self-categorization and gains prominence depending on intergroup context dynamics and global threats common to all humanity. Empirical evidence shows that this superordinate entity fulfills important functions: it has been observed that it can contribute to self-efficacy (Mohamed, 2025), as well as to the adoption of positive attitudinal responses toward planetary sustainability (Jeong and Kim, 2025) and contribution to the common good (Buchan et al., 2011). It has even been argued that it could mitigate intergroup differences and rivalries (see Penwell, 2018). Previous studies suggest that global identity, in addition to being related to universalistic values, may also be associated with self-transcendent values and emotions (Basabe et al., 2018; de Rivera, 2018; McFarland et al., 2019). Thus, in the present model, global identity represents the superordinate level of self-categorization whose situational salience is examined, whereas perceived threat is treated as a contextual condition that may alter the relevance of this level of identification.

### 1.2 Self-transcendent emotions

Self-transcendence is a subjective phenomenon that can contribute to self-awareness and openness to elements beyond one's individual existence, facilitating connection with entities broader than the self (Keltner and Haidt, 2003; Reed, 2010). Traditional psychologists conceptualized self-transcendence as the essence of human existence, a relevant human phenomenon in positive social functioning, and as a capacity to foster connection with others (Frankl, 1966; May, 1958). It was even conceived as a human need by Abraham Maslow in his hierarchy, when he established the subjective experience of peak experiences as one of the basic individual needs for human motivation (Maslow, 1964, 1970). More contemporary authors, such as Emmons (2005), state that self-transcendence is reflected in spiritual beliefs related to connection with others and the world, providing meaning and purpose in life (Peterson and Seligman, 2004; Van Cappellen et al., 2013). These connecting beliefs link the individual to broader social entities, such as humanity itself (Keltner and Haidt, 2003), providing a broader awareness of the world and a greater connection with others. In this sense, self-transcendent emotional experiences are defined as transient affective states that partially shift attention from the individual self toward other people or experiences of moral connection, decreasing self-absorption and increasing feelings of connection with the social environment (Stellar et al., 2017; Yaden et al., 2017).

Self-transcendent emotions represent a class of emotions that connect individuals with concern for the wellbeing of the whole (Haidt, 2003; Fredrickson, 2009; Van Cappellen et al., 2013; Van Cappellen and Saroglou, 2012). Unlike other positive emotions that are more centered on individual wellbeing, these emotions seem to foster an orientation toward interdependence, prosociality, and concern for others (Pizarro et al., 2021). Authors such as Abatista and Cova (2023) have established a taxonomy that classifies the functions of self-transcendent emotions into two major dimensions: epistemic states and social states. The former refers to the acquisition of awareness about oneself and the world through the self-transcendent emotional experience. They imply an evaluation of one's own thought processes and play a fundamental role in attention, motivation, and individual behavior (López-Cassà and Alzina, 2023). The latter orient thought and action toward other people, activating the moral component centered on an empathic or gratitude-based affective response, and evoke a connection with larger entities such as nature and the human species (Van Cappellen and Rimé, 2014; Van Cappellen et al., 2013).

In this sense, self-transcendent emotions can offer a relevant affective pathway for understanding how some people broaden their identification focus toward inclusive social categories such as humanity or the global community. In the context of this study, self-transcendent emotions are not conceived as a direct and sufficient cause of global identity, but rather as an emotional process that could accompany or facilitate the activation of broader forms of social belonging in situations of shared threat. Experimental evidence shows that self-transcendent emotional experience can increase superordinate identification by reducing self-focus and strengthening perceived interconnectedness (Seo et al., 2022), while recent field evidence also links self-transcendent emotions with broader social categories (Rincón-Unigarro et al., 2025). In the context of this study, self-transcendent emotions are not conceived as a direct and sufficient cause of global identity or as a component of Social Identity Theory itself, but as a complementary affective process that could accompany or facilitate orientation toward broader forms of social belonging in situations of shared threat.

### 1.3 The present study

This study examines the role of self-transcendent emotional experience in the relationship between perceived threat toward COVID-19 pandemic and global identification among the adult population in Chile.

The Chilean context provides a particularly informative setting for examining this relationship because the global threat posed by COVID-19 coincided with a highly salient domestic sociopolitical process marked by political uncertainty, social mobilization, and episodes of violence. While the pandemic represented a threat shared across national boundaries, the 2019 social outburst and subsequent constitutional process placed fundamental questions about Chile's political and institutional order at the center of public life. The plebiscite involved the possibility of replacing the 1980 Constitution, adopted during the Pinochet dictatorship, and opened a broader debate about the institutional foundations of the country and the future role of the state (Meléndez et al., 2021; Sáez-Vergara et al., 2022). This convergence is relevant for the study of global identity, although these sociopolitical conditions are considered here as contextual features rather than directly measured identity processes. Previous research on superordinate identification has often focused on either the pandemic as the focal collective threat or broader globalization processes, while giving comparatively less attention to contexts in which these dynamics unfold alongside a salient domestic sociopolitical crisis (Reysen et al., 2020; Hamer et al., 2024).

Using a longitudinal design with two measurements (T1-T2), responses obtained in September 2020 (T1) and November 2020 (T2) were analyzed. During these periods, Chile was not only facing a global pandemic crisis but was also immersed in significant social and political processes with implications for the nation-state. Regarding the health crisis, although in the first data collection (T1, September 2020) the country had already passed the peak of first-wave infections, official health indicators at that time reported a total of 462,991 accumulated cases and 12,741 deaths (Ministerio de Salud, 2020a), with the lethal threat of SARS-CoV-2 persisting. The second data collection (T2 November 2020) took place under greater control of the virus by authorities, showing significant improvement in health indicators compared to the previous date (Ministerio de Salud, 2020b).

In parallel, Chile was undergoing a deep sociopolitical process. September 2020 (T1) was a month of high national symbolic significance, coinciding with the celebration of national holidays (September 18) which commemorate Chile's independence. Also, during this month, a broad nationwide campaign (Approve vs. Reject) regarding the constitutional plebiscite to be held on October 25, 2020, was developed in a context of high uncertainty and violence due to the anniversary of the ‘Social Outbreak' of October 2019 (Johnson Barella, 2023; López-Escarcena et al., 2025; Sáez-Vergara et al., 2022). In addition, Chile experienced unfavorable macroeconomic indicators due to the pandemic, with unemployment rates above 10% (see Meléndez et al., 2021), causing labor and economic uncertainty among the population.

Within the social identity approach, these concurrent global and national conditions are relevant because the salience of social identities depends on the comparative and contextual conditions in which self-categorization occurs (Turner et al., 1987; Hornsey, 2008). Accordingly, perceived threat is treated here as a contextual condition that may make a superordinate human category more salient when the threat is experienced as shared across humanity (Hamer et al., 2024), whereas self-transcendent emotions are considered a complementary affective process that may facilitate orientation toward this broader level of identification (Seo et al., 2022; Rincón-Unigarro et al., 2025).

In this scenario, the study aims to address three main objectives: First, to examine whether perceived threat is associated with global identity through self-transcendent emotions at each measurement point. Second, to assess whether these associations show prospective evidence between T1 and T2 after controlling for the temporal stability of the constructs. Third, to analyze the longitudinal invariance of the global identity measure, to determine whether comparisons between measurements reflect ontogenetic changes in the construct and not variations in the psychometric functioning of the scale.

## 2 Materials and method

### 2.1 Participants and procedure

Participants were 224 adults (age *M* = 39.25, SD = 12.76; range = 18–71 years). Of the sample, 49.6% were women, 48.2% were men, and 2.2% identified as other. Regarding educational level, 0.9% had completed primary education, 21.4% secondary education, 58.5% undergraduate education, 7.6% postgraduate education, and 1.3% held a doctoral degree, while 10.3% were still studying. In terms of marital status, 41.5% were single, 12.5% were in a couple, 40.2% were married, 0.4% were widowed, and 5.4% were divorced.

Regarding the proportion of missing data, in T1, the total percentage of unanswered items was 0.20%, and only 9 participants (4.0%) had any unanswered questions, with a maximum of 3 unanswered questions per person. In T2, no missing values were recorded, so the information retention rate was 100%. The questionnaires were administered via online surveys created on the Survey Monkey^®^ platform. The services of Offer wise, a research panel company that provides programming, collection, processing, and data analysis (website: https://www.offerwise.com/), were contracted. Data collection was conducted during the COVID-19 pandemic, when strict mobility and face-to-face contact restrictions were in place in Chile, making in-person administration unfeasible. Offer wise applied its standard quality-control procedures for online data collection, and the research team closely supervised the implementation of the study protocol. At T1, participants were asked to provide an email address and to indicate whether they authorized the research team to contact them again for the T2 assessment.

Each participant was assigned an alphanumeric code, which was used to match their T1 and T2 responses while allowing the analytical dataset to be anonymized. At T2, only participants who had previously provided such authorization were contacted by email. All participants gave consent after being informed about the study, in which the voluntary nature of their participation and the confidentiality of their information were made explicit. The procedure was approved by the Ethics Committee of the Universidad Católica del Norte (Ref.: 0041/2019).

### 2.2 Instruments

To measure the perceived threat toward COVID-19 pandemic, the original scale by Pizarro et al. (2020) was used, constructed *ad hoc* and previously applied in different cultural contexts. This scale was considered appropriate because it captures both infection-related risk and concerns about the broader consequences of the pandemic, two central dimensions of perceived threat in the COVID-19 context (Pizarro et al., 2020, 2024). The instrument consists of seven items, which aim to capture the subjective degree of threat related to infection risk (“I am afraid of getting coronavirus”) and the uncertainty about possible social consequences of the pandemic crisis (“I am worried about losing my job or someone close to me losing theirs”). The prompt was: “Please indicate to what extent you agree with the following concerns related to the coronavirus pandemic:” The scale is a 7-point Likert-type (1 = Strongly disagree to 7 = Strongly agree), where higher scores indicate a greater degree of individual perceived threat regarding the COVID-19 pandemic. The internal consistency of the scale in our data was acceptable in both T1 and T2 (McDonald ω: 0.74–0.74).

To evaluate the self-transcendent emotional experience, self-transcendent emotions were measured using items that primarily refer to awe/wonder and moral elevation. Fourteen items were used, drawn from the original scales of Cusi et al. (2018) and Pizarro et al. (2018), both validated in Spanish, which capture these two emotional states. The prompt was: “Now, please answer the following questions, keeping the context of the pandemic in mind. Using a scale from 1 to 7 (1 = Not at all and 7 = Very much), indicate the intensity with which you experienced these reactions:” High scores indicate greater self-transcendent emotional experience. The internal consistency of the set of scale items showed acceptable levels at both measurement points (McDonald ω: 0.92 T1–0.93 T2). Given the above, the empirical conclusions of this research should be interpreted in relation to the specific operationalization of the self-transcendent emotions analyzed in this study—namely, awe/wonder and moral elevation—even though the theoretical-conceptual framework is situated within the broader self-transcendent emotion literature.

To measure global identity, the original scale by Der-Karabetian and Ruiz (1997) was used, which has been cross-culturally employed (de Rivera and Carson, 2015) and has shown acceptable psychometric properties in Spanish (see Alfaro-Beracoechea et al., 2019). The prompt was: “Please indicate to what extent the following statements correspond to your personal opinions and beliefs.” The instrument consists of seven items, with higher scores indicating greater global identification. Internal consistency also showed acceptable levels in both T1 and T2 (McDonald ω: 0.88–0.89). This scale captures an affective form of global-human identification centered on the feeling of belonging to a broad human community. Therefore, while it is conceptually linked to the literature on identification with all humanity and global citizenship, it should not be considered fully equivalent to these constructs, as it does not explicitly capture moral or normative content.

Table 1 presents the descriptive statistics (means, standard deviations, skewness, and kurtosis) of the three main scales at each of the two measurement points. Scores were calculated as the average of the items comprising each scale. In the total sample (*N* = 224), moderate levels of perceived threat (T1: *M* = 4.20, *SD* = 1.32; T2: *M* = 4.17, *SD* = 1.28), self-transcendent emotions (T1: *M* = 4.46, *SD* = 1.26; T2: *M* = 4.42, *SD* = 1.29), and global identity (T1: *M* = 4.25, *SD* = 0.98; T2: *M* = 4.09, *SD* = 1.01) were observed. Skewness and kurtosis values remained within acceptable limits (Kline, 2023), indicating distributions close to normal.

**Table 1:** Descriptive statistics of main variables (N = 224).

Variable	*M*	*SD*	Min	Max	Skewness	Kurtosis
RP T_1_	4.20	1.32	1.00	7.00	0.10	−0.41
RP T_2_	4.17	1.28	1.00	7.00	−0.01	−0.36
STE T_1_	4.46	1.26	1.23	7.00	−0.15	−0.21
STE T_2_	4.42	1.29	1.08	7.00	−0.02	−0.47
GI T_1_	4.25	0.98	1.00	6.00	−0.39	−0.21
GI T_2_	4.09	1.01	1.00	6.00	−0.63	0.55

RP, perceived threat; STE, self-transcendent emotions; GI, global identity. T_1_ = september 2020, T_2_ = november 2020. *M*, mean; *SD*, standard deviation; Min, minimum score; Max, maximum score.

### 2.3 Data analysis

To optimize the ratio between sample size and the number of parameters to be estimated (N:q), as well as improve the stability of factorial solutions, an item parceling strategy was applied to the original scales for each construct and time point (T1–T2). Three balanced parcels were created, assigning items so that each parcel included a similar number of indicators and, as far as possible, related content. All parcels were calculated as the average of their constituent items, allowing missing data to be managed through the average of available scores. Subsequent analyses used these parcels as indicators of the latent variables. The goodness of fit of all models was evaluated using robust indices: the robust comparative fit index (robust CFI), the robust root mean square error of approximation (robust RMSEA) with its 90% confidence interval, and the standardized root mean square residual (SRMR). Following the recommendations of Hu and Bentler (1999), values of CFI ≥0.90 (preferably ≥0.95), RMSEA ≤ 0.08 (preferably ≤ 0.05), and SRMR ≤ 0.08 were considered indicative of acceptable fit. The significance of indirect effects and latent mean difference was determined using the 95% BCa confidence interval, with the null hypothesis rejected when the interval did not include zero. Analyses were conducted in the R statistical environment (R Core Team, 2024) using the lavaan (Rosseel, 2012) and semTools (Jorgensen et al., 2026) packages.

## 3 Results

The correlation analyses showed a positive and statistically significant association between the study variables across both time points, with a tendency toward greater robustness at T2. Among all associations, those between self-transcendent emotions and global identity at both time points stood out for their considerable effect size (*r* >0.50), although the associations between perceived threat and self-transcendent emotions at both times showed a medium effect size (*r* >0.30) (Table 2). Consistent with the rest of the performed analyses, the observations were made using bootstrapping with 5,000 resamples, and the reported confidence intervals were calculated using the bias-corrected and accelerated (BCa) method.

**Table 2:** Pearson correlations (bilateral), confidence intervals, bootstrap BCa and levels of signification.

Variable	1	2	3	4	5	6
1. RP T_1_	1					
2. RP T_2_	0.583^***^ [0.466, 0.672]	1				
3. STE T_1_	0.303^***^ [0.158, 0.420]	0.243^***^ [0.108, 0.362]	1			
4. STE T_2_	0.239^***^ [0.101, 0.360]	0.332^***^ [0.192, 0.455]	0.567^***^ [0.452, 0.660]	1		
5. GI T_1_	0.214^**^ [0.092, 0.326]	0.177^*^ [0.058, 0.298]	0.559^***^ [0.447, 0.646]	0.309^***^ [0.177, 0.427]	1	
6. GI T_2_	0.183^**^ [0.048, 0.308]	0.284^***^ [0.158, 0.401]	0.426^***^ [0.271, 0.548]	0.528^***^ [0.412, 0.625]	0.575^***^ [0.466, 0.664]	1

*N* = 224. RP, perceived threat, STE, self-transcendent emotions; GI, global identity. T_1_ = september 2020, T_2_ = november 2020. ^***^ *p* < 0.001, ^**^ *p* < 0.01, ^*^ *p* < 0.05. Confidence Interval [CI 95%], BCa 5,000 bootstrapping.

### 3.1 Concurrent mediation model (T1 and T2)

To examine the concurrent associations between perceived threat, self-transcendent emotions, and global identity at each measurement point (T1 and T2), a structural equation model that integrates both time points was specified. The model included six latent factors (perceived threat, self-transcendent emotions, and global identity at T1 and their counterparts at T2), each defined by three respective parcels. The structural effects comprised the pathways from perceived threat to self-transcendent emotions (a), from self-transcendent emotions to global identity (b), and the direct effect of perceived threat on global identity (c), both at T1 and T2.

Longitudinal co-variances between homologous factors at T1 and T2 were also included to model the constructs' temporal stability. Estimation was performed using the maximum likelihood estimator (ML). Standard errors and confidence intervals were obtained via non-parametric bootstrap with 5,000 resamples, calculating bias-corrected and accelerated (BCa) 95% confidence intervals. For indirect effects to be considered significant, the intervals must not include zero. Missing values were handled using full-information maximum likelihood (FIML), assuming a missing-at-random mechanism. The missing data patterns revealed that omissions were sporadic and without any systematic structure, suggesting a completely random data loss mechanism (MCAR). Although the impact of this missingness on the results is negligible, model estimation allowed the use of the entire sample (*N* = 224) in all analyses.

The model showed a good fit: χ*2* (126) = 249.07, *p* < 0.001; robust CFI = 0.952; robust TLI = 0.941; robust RMSEA = 0.062 [90% CI (0.049, 0.075)]; SRMR = 0.053. The factor loadings of the parcels were all statistically significant (standardized λ between 0.55 and 0.91, and *p* < 0.001), confirming an adequate measurement of the latent constructs. The standardized and unstandardized coefficients, along with their BCa confidence intervals, are presented in Table 3.

**Table 3:** Standarized and non-standarized regression coefficients of concurrent mediational model.

Parameters	T_1_			T_2_		
	*B* (*SE*)	β	*95% BCa CI*	*B*(*SE*)	β	*95% BCa CI*
RP → STE (a)	0.38 (0.08)	0.35	[0.22, 0.56]	0.35 (0.09)	0.35	[0.19, 0.53]
STE → GI (b)	0.36 (0.05)	0.59	[0.27, 0.47]	0.36 (0.05)	0.56	[0.26, 0.47]
RP → GI (*c'*)	0.05 (0.05)	0.08	[−0.04, 0.15]	0.10 (0.05)	0.16	[0.02, 0.21]
Indirect effect (a^*^b)	0.14 (0.03)	0.21	[0.08, 0.22]	0.13 (0.04)	0.20	[0.07, 0.21]
*R^2^* STE	0.125			0.125		
*R^2^* GI	0.389			0.403		

RP, perceived threat; STE, self-transcendent emotions; GI, global identity. *B* = non-standarized coefficient. *SE*, standard error. β = standarized coefficient. Confidence Interval [CI 95%], BCa 5,000 bootstrapping.

In the first measurement (T1), perceived threat positively predicted self-transcendent emotions (a = 0.354, *p* < 0.001), and self-transcendent emotions, in turn, were positively associated with global identity (b = 0.590, *p* < 0.001). The direct effect of perceived threat on global identity was not statistically significant (c' = 0.081, *p* = 0.241), whereas the indirect effect was significant (ab = 0.209, *p* < 0.001). In the second measurement (T2), the associations were replicated: a = 0.353, *p* < 0.001; b = 0.561, *p* < 0.001. However, unlike T1, the direct effect was small but statistically significant (c' = 0.159, *p* = 0.026). The indirect effect at T2 was also statistically significant, with a magnitude similar to that observed at T1 (ab = 0.198, *p* < 0.001). This indicates that the association between perceived threat and global identity is not explained solely by the indirect pathway through self-transcendent emotions, as it also maintained a direct link at T2.

The longitudinal covariances between the latent factors across both measurement time points (T1 and T2) were high (perceived threat: ϕ = 0.75; self-transcendent emotions: ϕ = 0.60; global identity: ϕ = 0.67), indicating stability between the two time points. The proportion of variance explained in global identity was 38.9% at T1 and 40.3% at T2, while perceived threat explained 12.5% of the variance in self-transcendent emotions at both times. These results show a consistent indirect association in both measurements, with the indirect effect of self-transcendent emotions remaining between β = 0.20 and β = 0.21, and suggest that, at a concurrent level, perceived threat is related to higher global identity through the increase of self-transcendent emotions.

### 3.2 Half-longitudinal model (CLPA)

To assess the possible existence of prospective mediation, a two-wave cross-lagged panel model (CLPA) was estimated using the same parcel-based latent factors. The model included: (a) the autoregressive paths from each factor at T1 to T2; (b) the cross-lagged paths of interest (perceived threat T1 → self-transcendent emotions T2, self-transcendent emotions T1 → global identity T2, and perceived threat T1 → global identity T2); (c) the prospective indirect effect; and (d) longitudinal residual correlations between each parcel at T1 and its corresponding parcel at T2, to control for the specific variance of indicators over time. Estimation and the calculation of confidence intervals followed the same procedure described for the concurrent mediation model (ML, 5,000 bootstrap resamples, 95% BCa intervals, FIML).

The model fit indices were good: χ*2* (114) = 160.16, *p* = 0.003; CFI = 0.980; TLI = 0.973; RMSEA = 0.043 (90% CI (0.026,0.057)]; SRMR =0.047. The autoregressive stability coefficients were statistically significant (perceived threat: β = 0.70, *p* < 0.001; self-transcendent emotions: β = 0.57, *p* < 0.001; global identity: β = 0.59, *p* < 0.001), indicating that the constructs underwent minimal relative changes between T1 and T2. By contrast, none of the cross-lagged paths reached statistical significance (Table 4). All confidence intervals for the cross-lagged paths included zero, indicating the absence of significant prospective effects among perceived threat, self-transcendent emotions, and global identity within the analyzed interval. Perceived threat at T1 did not predict changes in self-transcendent emotions at T2 [β = 0.04, 95% BCa CI (−0.09, 0.16), *p* = 0.579]. Self-transcendent emotions at T1 also did not predict changes in global identity at T2 [β = 0.06, 95% BCa CI (−0.08, 0.19], *p* = 0.574]. The direct path from perceived threat T1 to global identity T2 was not statistically significant either [β = 0.06, 95% BCa CI (−0.06, 0.17), *p* = 0.455].

**Table 4:** Half-longitudinal model coefficients (CLPA).

Parameter	*B*	*SE*	*p*	*p*	95% BCa CI
Stability					
RP T_1_ → RP T_2_	0.752	0.11	0.70	< 0.001	[0.51, 0.88]
STE T_1_ → STE T_2_	0.559	0.07	0.57	< 0.001	[0.41, 0.71]
GI T_1_ → GI T_2_	0.595	0.10	0.59	< 0.001	[0.40, 0.80]
Cross paths					
RP T_1_ → STE T_2_	0.043	0.08	0.04	0.579	[−0.09, 0.16]
STE T_1_ → GI T_2_	0.039	0.07	0.06	0.574	[−0.08, 0.19]
RP T_1_ → GI T_2_	0.039	0.05	0.06	0.455	[−0.06, 0.17]

RP. perceived threat; STE, self-transcendent emotions; GI, global identity. T_1_ = september 2020, T_2_ = november 2020. *B* = non-standarized coefficient. *SE*, standard error. β = standarized coefficient. Confidence Interval [CI 95%], BCa 5,000 bootstrapping.

These results contrast with the robust and statistically significant indirect effects observed in the concurrent model (β = 0.21 at T1 and β = 0.20 at T2). Taken together, the absence of significant cross-lagged paths and the high temporal stability of the constructs suggest that the associations among perceived threat, self-transcendent emotions, and global identity were clearer at the concurrent level than at the prospective level. This pattern could reflect relatively stable differences between participants, a situated affective-identity configuration, or limitations of the two-wave design in capturing more dynamic change processes.

### 3.3 Longitudinal measurement invariance

To provide evidence that differences in latent means reflect genuine changes in the construct rather than psychometric artifacts, the measurement equivalence of global identity over time was examined. Levels of configural invariance (same factor structure, no constraints), metric invariance (equivalent factor loadings), and scalar invariance (equivalent loadings and intercepts) were tested on the original scale items. The models were estimated using the robust MLR estimator to correct possible deviations from multivariate normality. For the comparison of nested models, two complementary criteria were used: (a) the difference in robust CFI and robust RMSEA, considering a substantial deterioration in fit when increases are ΔCFI ≥0.010 and ΔRMSEA ≥0.015 (Chen, 2007); and (b) the likelihood ratio test with the Satorra-Bentler correction.

As shown in Table 5, the configural model (without constraints) showed an adequate fit. Imposing equality of factor loadings (metric invariance), and subsequently equality of intercepts (scalar invariance), did not substantially deteriorate the fit according to the changes observed in CFI and RMSEA. According to the recommended cut-off points (Chen, 2007), the differences are below the thresholds (|ΔCFI| < 0.010 and ΔRMSEA < 0.015): the difference in χ^2^ is not statistically significant for either metric invariance (*p* = 0.596) or scalar invariance (*p* = 0.101), and the ΔCFI and ΔRMSEA are minimal. Therefore, the results support the longitudinal scalar invariance of the global identity measure, allowing for the comparison of its latent means over time as a reflection of genuine ontogenetic change in the construct.

**Table 5:** Global Identity longitudinal measurement invariance.

Model	$\chi^{2}\text{S}-\text{B}\left(\text{gl}\right)$	CFI_robust_	RMSEA_robust_	SRMR	Comparison	$\Delta \chi^{2}\text{S}-\text{B}\left(\text{Δ}gl\right)$	*p* (Δ)	ΔCFI	ΔRMSEA
Configural	133.16 (69)	0.947	0.074	0.045	–	–	–	–	–
Metric	139.27 (75)	0.948	0.070	0.049	vs. configural	4.60 (6)	0.596	+0.001	−0.003
Scalar	151.48 (82)	0.945	0.069	0.054	vs. metric	11.98 (7)	0.101	−0.003	−0.001

*Note*. $\chi_{\text{S}-\text{B}\left(\text{gl}\right)}^{2}$ = robust chi-square statistic (Satorra-Bentler). $\Delta \chi_{\text{S}-\text{B}}^{2}$ = robust chi-square difference. The critical thresholds are |ΔCFI| ≥. 010 and ΔRMSEA ≥. 015 for relevant deterioration (Chen, 2007). All models were estimated using MLR and the residual longitudinal correlations between peer items were analyzed.

### 3.4 Latent means comparison

Once the level of scalar invariance was verified, the standardized difference between the latent means of global identity at T1 and T2 was estimated using a structured means model. In this model, the factor loadings and item intercepts were constrained to be equal over time: the factor mean at T1 was fixed at 0 and its variance at 1, so that the factor mean at T2 directly represents the standardized change, equivalent to a latent Cohen's d. The significance of this difference was evaluated using the 95% BCa confidence interval obtained from 5,000 bootstrap resamples.

The model fit was acceptable, although slightly lower than that of the invariance models without structured means: χ^2^S-B (89) = 212.06, *p* < 0.001, robust CFI = 0.902, robust RMSEA = 0.088. The CFI is at the conventional threshold of 0.90, while the RMSEA slightly exceeds the cut-off points established in the literature, suggesting a practically acceptable, though not optimal, fit. The change in global identity measure was small and statistically significant, *d* = −0.147, *SE* = 0.06, *p* = 0.014, 95% BCa CI [−0.264,−0.033]. The negative sign indicates that global identity decreased in the interval between T1 and T2. According to Cohen (1988) criteria, the effect size is modest, but the confidence interval excludes zero, providing evidence of a slight decrease in the latent mean of global identity between T1 and T2, although of limited magnitude (see Table 6).

**Table 6:** Differences in latent means and effect size.

Parameter	*d*	*SE*	*p*	95% BCa CI
Δ GI (T_2_ – T_1_)	−0.147	0.060	0.014	[−0.264,−0.033]

*N* = 224. GI, global identity. *d*, change in the latent mean (Cohen's d); SE, standard error. 95% confidence intervals are based on 5,000 bootstrap resamples with BCa correction. The mean and variance at T_1_ were set to 0 and 1, respectively, for identification purposes.

## 4 Discussion

This study examined the role of self-transcendent emotions in the association between perceived threat and global identity during the COVID-19 pandemic in Chile. The results showed concurrent indirect associations at both measurement points (T1 and T2): perceived threat was positively associated with self-transcendent emotions, which, in turn, were associated with higher levels of global identity. However, the cross-lagged model did not provide evidence of significant prospective effects after controlling for the temporal stability of the constructs. Additionally, the global identity scale showed evidence of longitudinal scalar invariance, allowing comparison of latent means and revealing a small decrease in global identity between the two measurements. This metric stability of the construct supports the interpretation of the observed difference in latent means as reflecting change in the construct rather than changes in its measurement properties (Chen, 2007; Cheung and Rensvold, 2002).

### 4.1 Self-transcendent emotions as a catalyst for superordinate categorization

The concurrent mediation analysis suggests that self-transcendent emotions may constitute a relevant affective process associated with superordinate identification, consistent with their capacity to reduce self-focus and expand the circle of social inclusion toward humanity (Pizarro et al., 2021; Seo et al., 2022). In the face of threat produced by the COVID-19 pandemic, self-transcendent emotions may therefore operate as an affective bridge compatible with greater salience of a common human identity, rather than demonstrating a recategorization from identities that were not directly measured in this study.

At T1, perceived threat was indirectly associated with global identity through self-transcendent emotions, whereas at T2 perceived threat also showed a small direct association with global identity (c' = 0.16), while the indirect association through self-transcendent emotions remained significant and similar in magnitude to that observed at T1 (β = 0.20). This pattern can be considered in light of social identity approaches emphasizing that broader levels of self-categorization may become psychologically relevant under particular contextual conditions (Gaertner et al., 1993; Hornsey, 2008; Leonardelli and Toh, 2015). However, the present findings do not establish that such a recategorization process occurred.

### 4.2 Discrepancy between concurrent associations and prospective models

The absence of significant cross-lagged effects introduces an important qualification to the concurrent findings. Although the associations among perceived threat, self-transcendent emotions, and global identity were observed at both measurement points, none of the cross-lagged paths reached statistical significance after controlling for autoregressive stability. The constructs showed substantial temporal stability across waves (β*s* = 0.57–0.70), which reduced the amount of residual variance available to be explained by prospective associations. Thus, the findings provide clearer evidence of relationships among the constructs within each measurement point than of prospective relationships across the interval examined.

Two interpretations should therefore be considered. First, the relationships among threat, emotion, and identity may be sensitive to the immediate context. From the perspective of Social Identity Theory and Self-Categorization Theory, the salience of social categories varies according to contextual conditions and does not necessarily imply a stable temporal sequence (Hornsey, 2008). Second, the relatively short interval between T1 and T2 may have limited the detection of prospective effects in relatively stable identity-related processes. Accordingly, the absence of cross-lagged effects should not be interpreted as evidence that the associations among perceived threat, self-transcendent emotions, and global identity are exclusively concurrent.

The Chilean context provides a relevant background for interpreting these associations. Although the first epidemic peak had passed and health indicators had improved by T2 (Ministerio de Salud, 2020a,b), pandemic-related economic, social, and institutional uncertainty remained salient in Chile (Henríquez Encamilla and Inostroza Jimenez, 2021; Meléndez et al., 2021). Chile's high uncertainty avoidance also provides a relevant cultural backdrop for considering the persistence of uncertainty during this period (Hofstede Insights, 2026). The coexistence of improving epidemiological indicators with continuing economic, institutional, and sociopolitical uncertainty may therefore help contextualize the associations observed within each wave. This interpretation remains tentative, however, because participants' subjective responses to these broader contextual conditions were not directly assessed.

### 4.3 Dynamics of global identity under social identity theory

The findings also showed a small but statistically significant decrease in the latent mean of global identity at T2 (*d* = −0.147). This change can be considered in light of Social Identity Theory and Optimal Distinctiveness Theory (Brewer, 1993), which emphasize that identification reflects a dynamic balance between inclusion and distinctiveness and may vary according to social and contextual conditions (Brewer, 2003; Leonardelli et al., 2010).

The Chilean cultural and sociopolitical context provides one possible framework for considering this small change. Chile's position on individualism–collectivism is compatible with sociological accounts in which individual agency coexists with interpersonal and collective ties (Hofstede Insights, 2026; Araujo and Martuccelli, 2014; Yopo, 2013), while the interval between T1 and T2 coincided with a highly salient constitutional campaign and plebiscite rooted in the 2019 social outburst (Johnson Barella, 2023; López-Escarcena et al., 2025; Sáez-Vergara et al., 2022).

Within Social Identity Theory and Self-Categorization Theory, the observed decrease is compatible with the possibility that the relative salience of the global category varied across this changing context. Recent evidence also indicates that self-transcendent emotional experience may be associated simultaneously with proximal and superordinate identities, with different strengths of association across levels of identification (Rincón-Unigarro et al., 2025). However, the decrease observed here should not be interpreted as evidence that participants shifted from global toward national or local identities, because these forms of identification were not directly assessed. The sociopolitical context therefore provides a possible interpretive framework rather than an established explanation for the change in global identity. Another interesting contextual result appears in the distribution pattern of global identity, where the observed changes in skewness and kurtosis reflect a divergent shift toward the extremes at T2. This change suggests that the distribution of global identity may have shifted between T1 and T2. However, these statistics do not allow identification of subgroups with distinct trajectories.

## 5 Conclusions

Taken together, this study highlights the relevance of self-transcendent emotional experience for understanding global identification in contexts of shared threat. At both measurement points, perceived threat was indirectly associated with global identity through self-transcendent emotions, indicating that the affective experience accompanying a global threat is relevant to understanding its relationship with highly inclusive forms of social identification. These findings complement social identity approaches by emphasizing the role of self-transcendent experience in the relationship between shared threat and superordinate identification.

The findings also show that the persistence of a global threat does not necessarily coincide with increasing global identification. During the period examined, global identity showed a small decline despite the continued presence of the COVID-19 pandemic. This result suggests that the relationship between global threats and superordinate identification is not straightforward and should be understood within the broader social context in which these experiences occur. The specific processes underlying changes in global identity, however, remain to be established.

## 6 Limitations and future research

This study has several limitations. First, the two-wave design does not permit a fully temporally separated test of the proposed indirect relationship among perceived threat, self-transcendent emotions, and global identity. In addition, the approximately two-month interval between T1 and T2 may have been too brief to capture substantive changes in relatively stable identity-related processes. Future studies with three or more measurement points and longer follow-up periods would allow a more adequate examination of these temporal dynamics (Cole and Maxwell, 2003).

Second, older adults were underrepresented, limiting generalizability to the broader adult population in Chile. The study also did not directly assess national or local identification, political orientation, engagement with the constitutional process, religiosity or spirituality, pandemic-related personal losses, or participation in collective events.

Future studies could examine the identification of subgroups with differentiated trajectories through analyses focused on individual change patterns or longitudinal profiles. Also, our results suggest the importance of examining whether the role of self-transcendent emotions varies according to the origin and nature of the perceived threat, the specific sociopolitical context in which the threat emerges, and broader cultural characteristics that could play a role in these processes, such as cultural values and belief systems. This could be especially relevant in non-WEIRD societies, such as Chile, where these phenomena have been partially studied.

## Data Availability

The raw data supporting the conclusions of this article will be made available by the authors, without undue reservation.
